# Hyperlipoproteinemia(a) and Severe Coronary Artery Lesion Types

**DOI:** 10.3390/biomedicines10112848

**Published:** 2022-11-08

**Authors:** Larisa N. Ilina, Olga I. Afanasieva, Andrey A. Shiryaev, Elina E. Vlasova, Said K. Kurbanov, Marina I. Afanasieva, Marat V. Ezhov, Vladislav P. Vasiliev, Damir M. Galyautdinov, Sergey N. Pokrovsky, Renat S. Akchurin

**Affiliations:** 1 A.L. Myasnikov Institute of Clinical Cardiology, Federal State Budgetary Institution National Medical Research Center of Cardiology Named after Academician E.I. Chazov, Ministry of Health of the Russian Federation, 121552 Moscow, Russia; 2 Institute of Experimental Cardiology Named after Academician V.N. Smirnov, Federal State Budgetary Institution National Medical Research Center of Cardiology Named after Academician E.I. Chazov, Ministry of Health of the Russian Federation, 121552 Moscow, Russia

**Keywords:** diffuse atherosclerosis, coronary artery calcinosis, coronary bypass grafting, lipoprotein(a)

## Abstract

Diffuse atherosclerosis and calcification of the coronary arteries (CA) create serious difficulties for coronary artery bypass grafting (CABG). The aim of this study was to compare demographic indicators, lipids, and clinical results one year after CABG in patients with different phenotypes of coronary artery (CA) disease. In total, 390 patients hospitalized for elective CABG were included in a single-center prospective study. Demographic data, lipids (total, low-density lipoprotein and high-density lipoprotein cholesterol, and triglycerides), and lipoprotein(a) (Lp(a)) concentrations were analyzed for all patients. Major adverse cardiovascular events (MACE) included myocardial infarction, stroke, percutaneous coronary intervention, and death from cardiac causes within one year after surgery. No significant outcome differences were found between the groups with diffuse vs. segmental lesions, nor the groups with and without calcinosis for all studied parameters except for Lp(a). Median Lp(a) concentrations were higher in the group of patients with diffuse compared to segmental lesions (28 vs. 16 mg/dL, *p* = 0.023) and in the group with calcinosis compared to the group without it (35 vs. 19 mg/dL, *p* = 0.046). Lp(a) ≥ 30 mg/dL was associated with the presence of diffuse lesions (OR = 2.18 (95% CI 1.34–3.54), *p* = 0.002), calcinosis (2.15 (1.15–4.02), *p* = 0.02), and its combination (4.30 (1.81–10.19), *p* = 0.0009), irrespective of other risk factors. The risk of MACE within one year after CABG was higher for patients with combined diffuse and calcified lesions vs. patients with a segmental lesion without calcinosis (relative risk = 2.38 (1.13–5.01), *p* = 0.02). Conclusion: Diffuse atherosclerosis and coronary calcinosis are associated with elevated Lp(a) levels, independent of other risk factors. The risk of MACE in the first year after surgery is significantly higher in patients with diffuse atherosclerosis and coronary calcinosis, which should be considered when prescribing postoperative treatment for such patients.

## 1. Introduction

Diffuse atherosclerosis and calcinosis of coronary arteries (CA) preclude effective myocardial revascularization and impede its benefit. In recent years, the proportion of patients with diffuse atherosclerosis and coronary calcinosis, which are often present concurrently, undergoing coronary artery bypass grafting (CABG) has significantly increased. In the current era, many patients undergo percutaneous revascularization, which can successfully delay surgical intervention. Microsurgical techniques, multiple arterial grafts, endarterectomies of coronary arteries, and extended distal anastomoses with shunt grafting may be used for more thorough surgical revascularization in such patients. Surgical approaches, including analysis of the immediate and long-term results of CABG in this cohort of “complicated” patients, remain the subject of many scientific studies. Improved surgical techniques and enhanced secondary prevention of CHD after successful surgery remain topical. We aimed to compare demographic parameters, including atherosclerosis risk factors and lipid profiles of patients with different types of coronary artery lesions, to the results 1 year after surgery in groups of patients with different atherosclerosis phenotypes.

Human plasma Lp(a) levels are genetically determined. When Lp(a) level exceeds 30 mg/dL (which is observed in approximately 30% of the human population), the risk of coronary atherosclerosis and its complications, as well as calcified aortic valve stenosis, increases linearly [1,2,3,4,5,6].

There is currently an increasing body of evidence concerning the involvement of Lp(a) in the processes of chronic inflammation in the vascular wall. Oxidized phospholipids covalently bound to one of the apolipoprotein(a) Kringle domains enhance arterial wall inflammation by activating circulating monocytes and endothelial cells, thereby contributing to the high atherogenicity of Lp(a) and initiating aortic valve calcinosis formation [7]. Lp(a) particles are found in atherosclerotic plaques and degenerative altered aortic valves and can directly participate in the pathogenesis of these diseases, contributing to endothelial dysfunction, lipid deposition, inflammation, and osteogenic differentiation in valve interstitial cells, leading to aortic valve calcification [8].

## 2. Materials and Methods

A single-center prospective study included 390 patients hospitalized for elective CABG surgery in 2018–2019. Patients enrolled in the study had clinical indications for CABG and no exclusion criteria. The type of coronary lesion (diffuse or focal) was based on the analysis of preoperative coronary angiographic data and intraoperative data, referring to both the diameter of the bypassed artery at the site of the anastomosis and the presence of calcification of the arteries targeted for bypass surgery. The syntax score was determined as described [9]. Patients with a diffuse type of lesion included those who had two or more target coronary arteries with a diameter of less than 1.5 mm distal to the lesion. Coronary calcinosis was defined as multiple persistent opacifications of the coronary wall visible in more than one projection surrounding the complete lumen of two or more target coronary arteries distal to the lesion according to cath data. The analysis of preoperative coronary angiography was performed independently by two interventional cardiologists. The analysis of intraoperative data was performed by a cardiothoracic surgeon. We intentionally used stronger criteria for diffuse coronary atherosclerosis to recognize patients with complex CAD, and the criteria of calcification were used according to the syntax definition. Demographic parameters (sex, age, the presence of type 2 diabetes mellitus (DM), arterial hypertension (AH), creatinine clearance (CC) (Cockcroft-Gault Equation), smoking, obesity (BMI ≥ 30 kg/m^2^) and overweight (BMI 25–29.9 kg/m^2^), past medical history (previous myocardial infarction (MI), percutaneous coronary intervention (PCI), peripheral artery disease (brachiocephalic and lower extremities)) were collected for all patients. The diagnosis of multifocal atherosclerosis was given in those cases when a patient had atherosclerotic plaques in brachiocephalic arteries or lower limb arteries narrowing the lumen by 50% or more. Concentrations of lipid spectrum parameters (total cholesterol (TC), low-density lipoprotein cholesterol (LDL C) and high-density lipoprotein cholesterol (HDL C), triglycerides (TG), and lipoprotein(a) (Lp(a))) were also determined for all patients. TC, HDL, and TG were determined on an Abbott analyzer (Abbott, USA), LDL concentration was calculated using the Friedewald formula, and corrected LDL cholesterol including cholesterol in Lp(a) (LDLcorr) was calculated using the same formula in Dalen modification [10]. To assess the results one year after the operation, a survey of the patients was conducted via phone calls to assess clinical events including MI, stroke, PCI, and cardiac death. Statistical analysis of the obtained data was performed using Microsoft Excel software and IBM SPSS Statistics, version 26. Before the quantitative data were analyzed, they were checked for normal distribution (Kolmogorov–Smirnov criterion). To compare parameters with normal distributions, the statistical methods of the *t*-test and one-factor analysis of variance were used, while the Mann–Whitney U-test was applied to compare parameters with non-normal or skewed distributions in the samples. The analysis of the correlation of qualitative characteristics in the groups was performed using the χ^2^ criterion. Prognostic model construction investigating the probability of a particular outcome was performed using multiple logistic regression. In order to assess the diagnostic significance of quantitative characteristics in predicting a particular outcome, the receiver operating characteristic curve analysis (ROC-analysis) method was employed. Differences were considered statistically significant at *p* < 0.05.

## 3. Results

The majority of patients (303 of 390) were men. The mean age of the patients was 64 ± 8 years. The youngest patient was 39 years old, while the oldest one was 80 years old. All patients had multivessel coronary lesions. Among all patients included in the study, 233 (60%) had diffuse lesions of coronary arteries and focal lesions were identified in 157 (40%). Widespread coronary calcinosis was detected in 70 patients or 18% of the total cohort of patients. Patients from groups with diffuse and segmental lesions, as well as with and without calcinosis (Table 1), had similar demographics. The mean age of patients in the groups with diffuse and segmental lesions was comparable (64.1 ± 7.4 vs. 63.5 ± 8.5 years, *p* = 0.65), as well as in the groups with and without calcinosis (63.8 ± 7.9 vs. 64.0 ± 7.7 years, *p* = 0.88). The estimated CC was also comparable in both types of CAD and in groups with calcinosis vs. the other groups. No differences were found in the incidence of high (>32) syntax scores between groups.

Comparing lipid parameters in patients with diffuse and segmental CA, levels of the Lp(a) concentration were significantly higher in patients with the diffuse type of CA lesions. In addition, there was a trend for a higher level of TC and LDL-C in the group with diffuse atherosclerosis. Lp(a) concentrations were also significantly higher in patients with severe coronary calcinosis, whereas other lipid levels did not differ (Table 2).

According to the results of the multivariate logistic regression analysis, elevated Lp(a) ≥ 30 mg/dL, hyperlipoproteinemia(a) [hyperLp(a)] OR = 2.21 (95% CI 1.35–3.60), *p* = 0.001, and increased LDL-Ccorr concentration at 38.7 mg/dL (1.31 (1.02–1.67), *p* = 0.03 were associated with the presence of diffuse CA lesions in patients, independently of their gender, age, presence of arterial hypertension, and DM type 2 or former smoking status. HyperLp(a) (OR = 2.22 (1.15–4.02), *p* = 0.01) and statin intake (0.38 (0.17–0.85), *p* = 0.02) were also related to the presence of CA calcinosis in a similar multivariable model.

**Figure 1:**
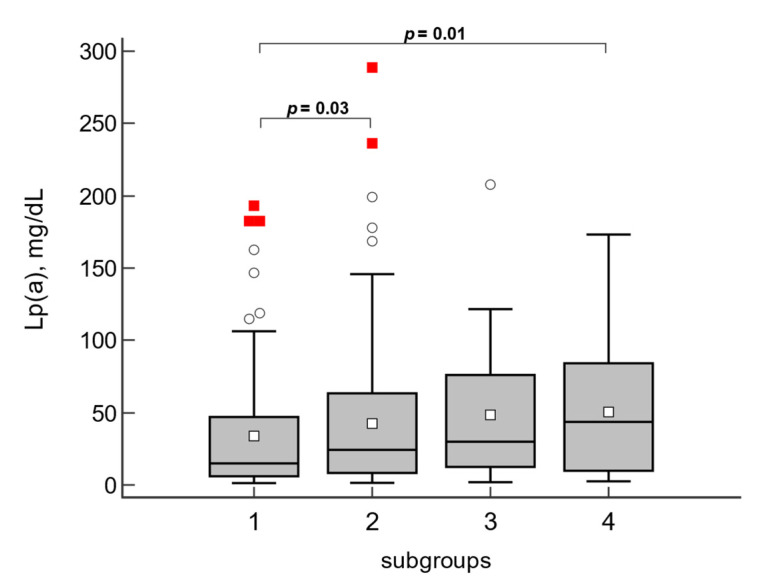
Lp(a) concentration in subgroups with different types of coronary arteries lesions. 1—segmental lesion without calcinosis, 2—diffuse lesion without calcinosis, 3—segmental with calcification, 4—diffuse with calcinosis. The data are presented as Box and Whisker graph. The circles indicate values outside the Q3 + 1.5 interquartile range (IQR), the red squares—values exceeding Q3 + 3 IQR.

When comparing Lp(a) levels of patients divided into four groups according to the combination of different atherosclerotic lesion phenotypes (segmental without calcinosis, diffuse without calcinosis, segmental with calcinosis, and diffuse with calcinosis), the highest level was observed in the most severe type (diffuse lesions with CA calcinosis) (Figure 1).

**Figure 2:**
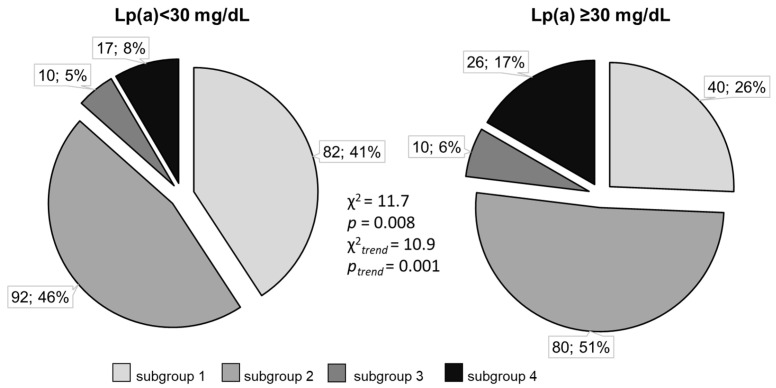
Distribution of different types of coronary artery lesions depending on the Lp(a) concentration. Subgroup 1—segmental lesion without calcinosis, subgroup 2—diffuse lesion without calcinosis, subgroup 3—segmental with calcification, subgroup 4—diffuse with calcinosis.

According to the ROC analysis, an Lp(a) concentration of more than 33 mg/dL was associated with the presence of diffuse CA lesions (sensitivity of 48%, specificity of 68%), with calcinosis of CA (sensitivity of 54%, specificity of 61%), and their combination (sensitivity of 60%, specificity of 70%). Compared to the patients with Lp(a) levels < 30 mg/dL, among patients with hyperLp(a), diffuse lesions with CA calcinosis were significantly more common, while segmental lesions without calcinosis were less common (Figure 2).

**Figure 3:**
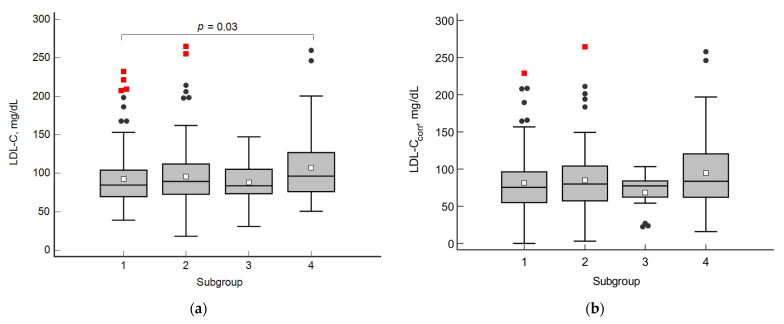
Concentration of LDL-C (**a**) and LDL-Ccorr (**b**) in subgroups of patients with different types of coronary artery lesions. 1—Segmental lesion without calcinosis, 2—diffuse lesion without calcinosis, 3—segmental with calcification, 4—diffuse with calcinosis. The circles indicate values outside the 1.5 IQR relative to the upper Q3 or lower Q1 quartiles; the red squares-values outside 3 IQR.

In the subgroup of patients with diffuse lesions and calcinosis, the level of LDL-C was significantly higher than that in patients with segmental lesions alone (Figure 3a), but the significance of differences disappeared when analyzing LDL-Ccorr, which takes into account the concentration of cholesterol in Lp(a) (Figure 3b).

An increased concentration of Lp(a) ≥ 30 mg/dL along with an increase in LDL-Ccorr by 38.7 mg/dL (1 mmol/L) and statin therapy were associated with the most complicated atherosclerotic lesion phenotype (diffuse lesion with calcinosis) both compared to patients with segmental lesions only and patients with any other lesion phenotypes (subgroups 1–3) (Table 3).

One year after surgery, 354 (91%) out of 390 patients participated in a telephone survey. In the group with diffuse coronary artery disease, 10 out of 205 patients had adverse outcomes (4.9%): Four experienced acute coronary syndrome with a fatal outcome, three had nonfatal MI (two of them with PCI), one had PCI due to recurrent angina pectoris, and two had undergone stroke. In the group of patients with the segmental type of coronary lesions (149 respondents), four people had adverse outcomes (2.7%): Two were fatal MI, one had non-fatal MI, and one had stroke. When categorized according to coronary artery calcinosis (65 respondents), four patients had unfavorable outcomes (6.2%): One had fatal MI, two experienced nonfatal MI (one of them with PCI), and one had PCI because of recurrent angina pectoris. Among those without coronary calcinosis (289 respondents), ten patients (3.5%) had adverse outcomes: five fatal MI, two non-fatal MI with PCI, and three cases of stroke. The maximal risk of MACE was observed in the subgroup of patients with a combination of diffuse lesions and coronary artery calcinosis (Table 4).

**Figure 4:**
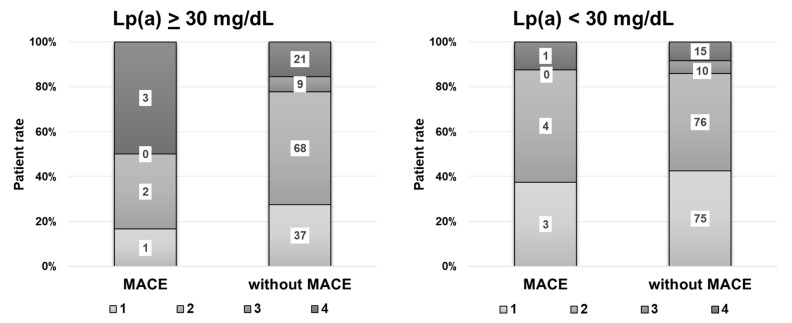
Proportion of patients with or without MACE in the first year after surgery. 1—Segmental lesion without calcinosis, 2—diffuse lesion without calcinosis, 3—segmental with calcification, 4—diffuse with calcinosis.

According to the correlation analysis and ROC analysis, Lp(a) concentration and other parameters of the lipid panel were not associated with the occurrence of MACE in patients in the first year after surgery in the total group of patients. However, among patients with hyperLp(a), there were more patients with diffuse lesions with calcinosis who experienced MACE than patients with normal Lp(a) levels (Figure 4).

## 4. Discussion

Diffuse lesions and coronary artery calcinosis create significant challenges for coronary surgeons. To conduct CABG in such patients, a complex surgical technique is used in combination with optical magnification, which is currently practiced in a limited number of cardiac surgery clinics. In some cases, a patient with such lesions is considered inoperable. Furthermore, patients with multivessel diffuse coronary artery lesions usually suffer from severe forms of CHD, including high-grade angina and chronic heart failure, because their coronary blood flow reserves are extremely limited. In order to identify factors associated with the development of severe coronary artery lesion phenotypes in patients referred for CABG, as well as to assess their relationship with adverse events one year after the operation, this study was undertaken.

When comparing demographic parameters, atherosclerosis risk factors, and the lipid spectrum in patients with diffuse and segmental lesion phenotypes, only differences in Lp(a) level were found to be significantly correlated. The median level in the group with diffuse lesions was 28 (8; 65) mg/dL, and in the group with segmental lesions, it was 16 (6; 47) mg/dL (*p* < 0.05). The same pattern was observed when comparing groups of patients with and without calcinosis of CA: The median Lp(a) level in the group of patients with calcinosis was 35 (11; 83) mg/dL, which was significantly higher than in the group of patients without calcinosis of CA—19 (8; 58) mg/dL (*p* < 0.05). HyperLp(a) was associated with both diffuse coronary artery lesions and their calcinosis independently of other atherosclerosis risk factors and lipid profile parameters. In the most severe lesions, diffuse atherosclerosis was combined with coronary calcinosis, and in this subgroup, the level of Lp(a) was the highest—44 (10; 83) mg/dL. All patients admitted for elective CABG surgery received lipid-lowering therapy with statins, in some cases in combination with ezetimibe, and the target level of LDL cholesterol (54 mg/dL or 1.4 mmol/L) was only achieved in 30 patients, which was approximately 8% of the total number of the patients. The contribution of cholesterol included in Lp(a) can significantly distort LDL-C, so for patients with an elevated concentration of Lp(a), it is reasonable to calculate LDL-Ccorr by the concentration of cholesterol included in Lp(a), rather than LDL-C [11,12]. Despite residual hypercholesterolemia, there were no significant differences in LDL-C levels in the groups compared with different phenotypes of atherosclerotic CA lesions. However, it should be noted that when comparing patients with diffuse and segmental lesions, there was a clear trend of higher levels of TC and LDL cholesterol in patients with diffuse atherosclerosis (159 (135; 189) vs. 150 (133; 174) mg/dL and 91 (73; 115) vs. 84 (70; 105) mg/dL), although the differences did not reach significance (*p* = 0.05 and *p* = 0.07). No such trend was observed when comparing traditional lipid risk factors for atherosclerosis in patients with and without CA calcinosis. The syntax score is a useful tool to determine the best surgical strategy, but it is mainly focused on anatomical predictors of the durability of PCI (not bypass grafts). The syntax score lacks the demonstration of coronary anatomy that limits CABG. It should be a reason for comparable scores in patients with complex CAD (diffuse atherosclerosis and calcinosis) and segmental lesions.

Based on our results, Lp(a) is involved in the development of the most severe phenotypes of atherosclerotic lesions of the CA. It is interesting to speculate whether this could be related to oxidized phospholipids included in Lp(a).

In our study, the analysis of MACE (MI, stroke, PCI, cardiac death) one year after CABG in groups with different phenotypes of coronary lesions showed a statistically significant increased risk in the group of patients with a combination of diffuse lesions and coronary calcinosis (RR = 2.38, *p* < 0.05). The data obtained in this study are consistent with a large Canadian study (802 patients after CABG), which proved that patients with a diffuse lesion and small diameter of bypassed arteries have worse long-term results (lower survival rate of 1.9 years after surgery) compared to patients with segmental lesion—84.5% vs. 92.1% [13].

Previously, we have shown that hyperLp(a) is associated with an increased risk of fatal outcomes and non-fatal MI in the long term after CABG surgery (5–10 years after the intervention) [14,15]. We also showed that Lp(a) levels were significantly higher in patients with one or more occluded vein grafts one year after CABG [16].

The use of a new class of drugs, named antisense oligonucleotides, which block apo(a) synthesis, resulted in a significant decrease in the concentration of Lp(a) and oxidized phospholipids and a decrease in inflammation and endothelial migration of monocytes [17] and may play a role in the further treatment of patients with hyperLp(a).

## 5. Limitations

The first limitation is the absence of a widespread definition of diffuse coronary artery disease; it is the reason our study is limited by the determination of lesion type. Furthermore, we cannot rule out possible subjectivity in the evaluation of coronary arteries. The next limitation is the sample size, which is common for a single-center study. However, it is notable that the results presented here have significant differences in laboratory and clinical outcomes. It is also important to note the limitations in gathering data. In particular, some patients could not attend the clinic, and information was collected through a telephone survey.

## 6. Conclusions

Our study showed an independent association of elevated Lp(a) concentrations with severe atherosclerotic phenotypes, including diffuse atherosclerosis and coronary artery calcinosis, as well as their combination, independent of other risk factors. One year after CABG, the risk of MACE was twice as high in patients with diffuse coronary artery disease and calcinosis compared to patients with segmental coronary artery disease. It seems relevant to study the long-term results of CABG surgery depending on the phenotype of coronary lesions and Lp(a) levels. The emergence of new pharmacological approaches for the substantial (80–90%) reduction of the Lp(a) concentration in the blood may improve the long-term prognosis of patients after CABG and deserves further investigation.

## Figures and Tables

**Table 1:** Demographics in patients with diffuse or segmental lesions and with or without coronary artery calcinosis.

	Type of Coronary Artery Disease			Severe Coronary Artery Calcinosis		
Parameters	Diffuse Lesions *n* = 233	Segmental Lesions *n* = 157	*p*	Yes *n* = 70	No *n* = 320	*p*
Gender (female)	51 (22%)	36 (23%)	0.81	11 (16%)	76 (24%)	0.18
Age	64.1 ± 7.4	63.5 ± 8.5	0.65	63.8 ± 7.9	64.0 ± 7.7	0.88
Obesity	101 (43%)	60 (38%)	0.35	30 (43%)	131 (41%)	0.79
Overweight	98 (42%)	78 (50%)	0.14	38 (54%)	138 (43%)	0.09
Smoking	90 (39%)	73 (46%)	0.14	28 (40%)	135 (42%)	0.79
Hypertension	217 (93%)	144 (92%)	0.70	68 (97%)	293 (92%)	0.13
MI	133 (57%)	97 (62%)	0.40	39 (56%)	191 (60%)	0.59
PCI	57 (24%)	46 (29%)	0.29	16 (23%)	87 (27%)	0.55
DM	60 (26%)	42 (27%)	0.91	22 (31%)	80 (25%)	0.29
CC	95 (77; 114)	96 (79; 111)	0.62	95 (74; 116)	96 (78; 112)	0.88
PAD Syntax Score > 32	65 (28%) 181 (78%)	42 (27%) 114 (73%)	0.82 0.25	23 (33%) 54 (77%)	84 (26%) 241 (75%)	0.30 0.75

Data are presented as *n* (%), mean ± standard deviations or median (me) and interquartile range (Q1; Q3). MI—myocardial infarction; PCI—percutaneous coronary intervention; DM—diabetes mellitus; CC—creatinine clearance; PAD—polyvascular atherosclerosis diseases.

**Table 2:** Lipids in patients with diffuse and segmental types of coronary artery lesions and with or without CA calcinosis.

	Type of Coronary Artery Disease			Coronary Artery Calcinosis		
Lipids	Diffuse Lesions *n* = 233	Segmental Lesion *n* = 157	*p*	Yes *n* = 70	No *n* = 320	*p*
TC, mg/dL	159 (135; 189)	150 (133; 174)	0.05	161 (141; 194)	154 (133; 181)	0.88
TG, mg/dL	120 (91; 155)	119 (91; 159)	0.72	120 (97;159)	120 (91; 156)	0.89
LDL-C, mg/dL	91 (73; 115)	84 (70; 105)	0.07	92 (75;119)	87 (71; 109)	0.40
LDL-Ccorr, mg/dL	80 (57; 106)	76 (55; 96)	0.21	79 (63;100)	78 (55; 102)	0.72
HDL-C, mg/dL	39 (33; 45)	40 (34; 45)	0.91	39 (32;45)	39 (34; 45)	0.67
Lp(a), mg/dL	28 (8; 65)	16 (6; 47)	0.02	35 (11; 83)	19 (8; 58)	0.046

Data are presented as median (me) and interquartile range (Q1; Q3). TC—total cholesterol, TG—triglycerides, LDL-C—low-density lipoprotein cholesterol, LDL-Ccorr—low-density lipoprotein cholesterol corrected for the concentration of Lp(a), HDL—high-density lipoprotein cholesterol, Lp(a)—lipoprotein(a).

**Table 3:** Results of multivariate logistic regression analysis of the relationship of hyperLp(a) and other risk factors with the presence of diffuse lesions with calcinosis of coronary arteries.

Risk Factors	Model 1	*p*	Model 2	*p*
Male gender	1.71 (0.64–4.55)	0.28	2.91 (0.91–9.33)	0.07
Age	1.03 (0.98–1.08)	0.28	1.05 (0.99–1.10)	0.11
Hypertension	3.68 (0.45–30.13)	0.22	4.06 (0.41–40.14)	0.23
Smoking	1.08 (0.67–1.74)	0.75	0.82 (0.45–1.48)	0.50
Lp(a) ≥ 30 mg/dL	2.69 (1.30–5.57)	0.008	4.29 (1.85–9.95)	0.0007
LDL-Ccorr ^#^	1.43 (1.05–1.95)	0.024	1.54 (1.07–2.24)	0.02
HDL-C ^#^	1.28 (0.26–6.11)	0.76	1.91 (0.25–14.45)	0.52
Diabetes mellitus	1.23 (0.56–2.70)	0.60	1.29 (0.50–3.35)	0.60
Statins	0.33 (0.14–0.79)	0.01	0.32 (0.11–0.99)	0.05

Data are presented as odds ratio (95% confidence interval). Model 1—subgroup 4 patients with diffuse coronary artery (CA) lesions and calcinosis vs. patients in subgroups 1–3; model 2—subgroup 4 patients with diffuse CA lesions and calcinosis vs. patients with segmental CA lesions and no calcinosis. ^#^ Increase of 38.7 mg/dL (1 mmol/L).

**Table 4:** The relative risk of adverse outcomes one year after surgery depending on the phenotype of coronary artery involvement.

Type of Coronary Artery Diseases	RR (95% CI)	*p*
Segmental	1	-
Diffuse	1.82 (0.58–5.68)	0.31
Without calcinosis	1	-
With calcinosis	1.78 (0.58–5.49)	0.32
Segmental without calcinosis	1	
Diffuse without calcinosis	1.34 (0.79–2.26)	0.27
Segmental without calcinosis	0.85 (0.06–12.21)	0.91
Diffuse with calcinosis	2.38 (1.13–5.01)	0.02

Data are presented as relative risk (95% confidential interval).

## Data Availability

Not applicable.
